# NADPH oxidase activator 1 (NOXA1) suppresses ferroptosis and radiosensitization in colorectal cancer

**DOI:** 10.7150/ijms.107038

**Published:** 2025-02-18

**Authors:** Qingyu Jiang, Qianping Chen, Quanquan Sun, Dong Liu, Ji Zhu, Wei Mao

**Affiliations:** 1 The Second School of Clinical Medicine, Zhejiang Chinese Medical University, Hangzhou 310053, China.; 2 Department of Radiation Oncology, Zhejiang Cancer Hospital, Hangzhou 310000, Zhejiang, China.; 3 Hangzhou Institute of Medicine (HIM), Chinese Academy of Sciences, Hangzhou 310000, China.; 4 Zhejiang Key Laboratory of Radiation Oncology, Hangzhou 310000, China.

**Keywords:** *Noxa1*, colorectal cancer, ferroptosis, radiotherapy sensitization, ROS

## Abstract

Radiotherapy is one of the main treatments for colorectal cancer (CRC), but due to the intrinsic resistance of cells or resistance caused by long-term radiotherapy, the effectiveness of this treatment is limited for some CRC patients. Consequently, identifying novel sensitization strategies is essential. This study identifies *Noxa1* as a marker linked to radiotherapy resistance in CRC, suggesting its potential as a prognostic biomarker for patients with CRC. The study found that *Noxa1* was significantly overexpressed in radiotherapy-resistant colorectal cancer patients, correlating with a poor prognosis. Additionally, we discovered that the high expression of *Noxa1* was negatively correlated with ferroptosis and primarily played a role through the glutathione metabolic pathway, as indicated by GSVA analysis. Experimental data indicated that the expression levels of NOXA1, SLC7A11, and GPX4 were significantly elevated in CRC cell lines resistant to radiotherapy. The expression of SLC7A11 and GPX4 decreased after the knockdown of *Noxa1*, leading to an increase in cellular ROS levels, which induced ferroptosis and sensitized the cells to radiotherapy. Therefore, *Noxa1* might influence the radiotherapy sensitivity of CRC via regulating ferroptosis. Targeting *Noxa1* could enhance radiotherapy sensitization and improve the prognosis of CRC patients.

## Background

Colorectal cancer ranks among the most prevalent malignancies worldwide, holding the third highest incidence and mortality rate globally^1^. In the management of CRC, radiotherapy is a crucial tool for addressing unresectable tumors, postoperative residual tumors, and recurrent tumors. Its benefits encompass primary tumor size reduction, tumor grade reduction, enhanced local tumor control, and the preservation of vital organs^2^-^4^. Nonetheless, the efficacy of radiotherapy can be limited in patients with inherently radiotherapy-resistant colorectal cancer or those who develop treatment-induced resistance^5^, ^6^. Radiotherapy for CRC can therefore be enhanced by identifying potential targets that can enhance the cells' radiosensitivity. Understanding the associated molecular mechanisms holds promise for significantly improving patient prognosis.

The NOXA1 enzyme is a key component of the activation of NOX1, a member of the NADPH oxidase family known to regulate ROS production^7^, ^8^. NOXA1 has been shown to play an important role in hypertension, vascular inflammation, stenosis, and atherosclerosis through its role in regulating ROS^9^-^11^. Notably, Epithelial-Mesenchymal Transition, hypoxia, angiogenesis, and activated inflammatory responses contribute to the poor prognosis of colorectal cancer, and NOXA1 has been found to be involved in vascular inflammation; thus, the expression of *Noxa1* may predict poor prognosis in CRC patients^12^.

Radiotherapy is based on ionizing radiation that directly or indirectly induces ROS production, resulting in DNA double-strand breaks that lead to tumor cell death^13^. Tumor cells adapt to oxidative stress by limiting ROS production or activating their antioxidant systems, a phenomenon linked to radiotherapy resistance^14^-^18^. ROS is a key regulator of ionizing radiation, and NOXA1 has the function of regulating ROS production, so our aim is to explore whether *Noxa1* influences the radioresistance of CRC by regulating ROS levels.

Ferroptosis, a modality of programmed cell death, characterized by iron accumulation and ROS generation, which catalyze lipid peroxidation of unsaturated fatty acids and decrease levels of glutathione peroxidase 4 (GPX4), thereby inducing cell death^19^-^23^. Increasing evidence indicates that tumor cells can upregulate SLC7A11 and GPX4 to protect cells from radiotherapy-induced ferroptosis, suggesting ferroptosis is crucial to tumor radiosensitivity^24^-^26^. As a result, targeting ferroptosis in radiotherapy may be a potential therapeutic strategy for overcoming radiotherapy resistance in tumors.

The study aimed to investigate the functional role of *Noxa1* in CRC radioresistance and its underlying mechanisms. Specifically, we explored the mechanism of interaction between *Noxa1* and ferroptosis in relation to radioresistance in CRC and explored the feasibility of using *Noxa1* as a biomarker for radioresistance.

## Materials and methods

### Identification of RTDEGs

Transcriptome data and associated clinical information were obtained from the Cancer Genome Atlas (TCGA) database. Raw gene expression counts were collected from the GSE35452 dataset in GEO. Differential gene expression analysis between CRC patients who responded to radiotherapy and those who did not was performed using the R package "limma" (version 4.2.1) with a standard comparison model. RTDEGs were identified with a threshold of absolute log2-fold change (FC) ≥ 0.3 and adjusted P-value < 0.05.

### Functional enrichment analysis of RTDEGs

Functional enrichment analysis of Gene Ontology (GO) and Kyoto Encyclopedia of Genes and Genomes (KEGG) pathways was carried out via the Metascape database (http://metascape.org/) to comprehensively understand the biological processes and pathways linked to RTDEGs. The top 20 critical signaling pathways were selected for further exploration (P<0.05).

### Survival analysis of RTDEGs

The median expression level of the *Noxa1* gene was utilized to establish an optimized cut-off value, stratifying colorectal cancer patients into high- and low-risk groups. We employed Kaplan-Meier survival curves and time-dependent ROC curves to demonstrate significant prognostic differences between the two groups and assess the *Noxa1* gene's efficacy as an overall survival (OS) biomarker.

### Predictive nomogram construction and evaluation

Univariate and multivariate COX analyses were conducted to assess *Noxa1*'s independent predictive ability in comparison to T stage, M stage, N stage, age, and gender (P < 0.05). For each factor, hazard ratios and their 95% confidence intervals were computed. A predictive nomogram was then established, combining the expression level of the *Noxa1* gene with clinical characteristics as a quantitative tool for predicting survival risk in colorectal cancer patients. Meanwhile, the accuracy of the nomogram's predictions was assessed using a calibration curve, where closer alignment with the 45° line indicates better predictive performance. The nomogram and calibration curve were generated using the "rms" R package.

### Correlation between ferroptosis and *Noxa1* expression

The ferroptosis score for each of the 64 ferroptosis regulator gene sets was calculated using the GSVA algorithm. This process utilized two input files: a gene set of 64 ferroptosis regulators and a standardized gene expression matrix of patient samples. We then analyzed the correlation between *Noxa1* and ferroptosis, as well as the relationships between *Noxa1* and key pathways such as iron ion metabolism, lipid metabolism, etc. Gene sets from the Molecular Signatures Database were analyzed using the GSVA package in R.

### Hematoxylin and Eosin (H&E) and immunohistochemistry (IHC) staining in CRC tissues

Colorectal cancer (CRC) samples were formalin-fixed, paraffin-embedded, and sectioned into 4-μm slices. For H&E staining, tissue sections underwent deparaffinization and rehydration before being stained with hematoxylin for 4 minutes and eosin for 90 seconds. The sections underwent dehydration using ascending ethanol concentrations, were cleared with xylene, mounted in neutral resin, and then examined microscopically. Immunohistochemistry (IHC) involved staining tissue sections using an anti-NOXA1 antibody, followed by hematoxylin counterstaining. IHC images were obtained at 400× magnification using a microscope, and semi-quantitative analysis of the staining was performed on three random fields with Image J software.

### Cell lines and culture conditions

Colorectal cancer (CRC) cell lines HCT8, HCT15, HCT116, and DLD-1 were sourced from the Institute of Biochemistry and Cell Biology, Chinese Academy of Sciences, Shanghai, China. The radio-resistant CRC cell lines HCT8R, HCT15R, HCT116R, and DLD-1R, were derived from their respective parental cell lines through 25 cycles of exposure to 2 Gy X-rays at a dose rate of 1 Gy/min. All cell lines were maintained in high-glucose DMEM (Gibco, USA) with 10% fetal bovine serum (Gibco, NY, USA) and 1% penicillin/streptomycin (Gibco, USA) under 5% CO_2_ and 95% air humidity. Regular testing for mycoplasma contamination was conducted every two months to ensure cell line integrity.

### Three-dimensional (3D) cell culture

A three-dimensional lrECM on-top culture was performed following established protocols. A 24-well plate was coated with 150 µl of Matrigel (Corning, NY, USA; 6347014). HCT15R and HCT8R cells were seeded onto the Matrigel layer (2×10^4^ cells per well), followed by the addition of medium with 10% Matrigel on top. Cells were incubated at 37°C for one week, with the medium replaced every 2-3 days. Colony cell numbers and the diameter of 3D cells were observed and analyzed using Image J software.

### Cell irradiation

CRC cells were subjected to varying radiation doses with an X-ray irradiator (SARRP III, Precision X-Ray, Inc, X Strahl, USA), operating at 13mA and employing 0.15mm copper for treatment and 1.0mm aluminum for imaging.

### Small interfering RNA (siRNA) transfection

CRC cells were transfected with 20μM *Noxa1*-specific siRNA using Lipofectamine 2000 (Invitrogen, 11668019), according to the manufacturer's instructions. Transfection efficiency was assessed by Western blot analysis 48-72 hours post-transfection. The siRNA sequences were as follows: si-*Noxa1* 1# (sense: 5′-ACC AUG AUG CCA GGU CCC UAA TT-3′, antisense: 5′-UUA GGG ACC UGG CAU CAU GGU TT-3′); si-*Noxa1* 2# (sense: 5′-CCA GCU UGG GCA ACU CAG UUA TT-3′, antisense: 5′-UAA CUG AGU UGC CCA AGC UGG TT-3′); si-*Noxa1* 3# (sense: 5′-CCU GCG GUU CAA GCU GCA ATT-3′, antisense: 5′-UUG CAG CUU GAA CCG CAG GTT-3′).

### CCK8 assay

Cell Counting Kit-8 (CCK-8) assays were performed according to the protocol provided by the manufacturer (Beyotime Biotechnology, Shanghai, China). In summary, 2000 cells in 100μl of culture medium were placed in each well of a 96-well plate and incubated for 1 to 7 days.

### Quantification of intracellular ROS levels

To evaluate the production of intracellular ROS, CRC resistant cells with si-*Noxa1* transfection underwent 6Gy X-ray irradiation. Six hours after irradiation, cells were collected, resuspended at 0.5×10^5^ cells/mL, and exposed to 10μM DCFH-DA fluorescence probe. The cell samples were incubated at 37°C for 30 minutes in the dark. The levels of mitochondrial ROS were visualized using a fluorescence microscope.

After 6 hours of irradiation, cells were incubated with the DCFH-DA fluorescence probe, diluted 1:1000 in serum-free medium, at 37°C for 30 minutes. The ROS levels were quantified using flow cytometry (Beckman, USA) and analyzed using FlowJo software. The results are expressed as mean percentages ± standard deviation (SD) of ROS-positive cells compared to the total cell population in the plots.

### Statistical analysis

Continuous variables are presented as the mean ± SD. Group differences were assessed using the Wilcoxon test in R software. Survival time variations were evaluated using the log-rank test, with a significance threshold set at P < 0.05. Kaplan-Meier plots were generated to visualize differences in survival time. Results are expressed as means ± SD from three independent experiments, and statistical analyses were conducted using GraphPad Prism 8.0c Software. Group differences were determined using the t-test, with statistical significance defined as a p-value < 0.05.

## Results

### Identification and functional annotation of RTDEGs associated with radiotherapy resistance

**Figure 1:**
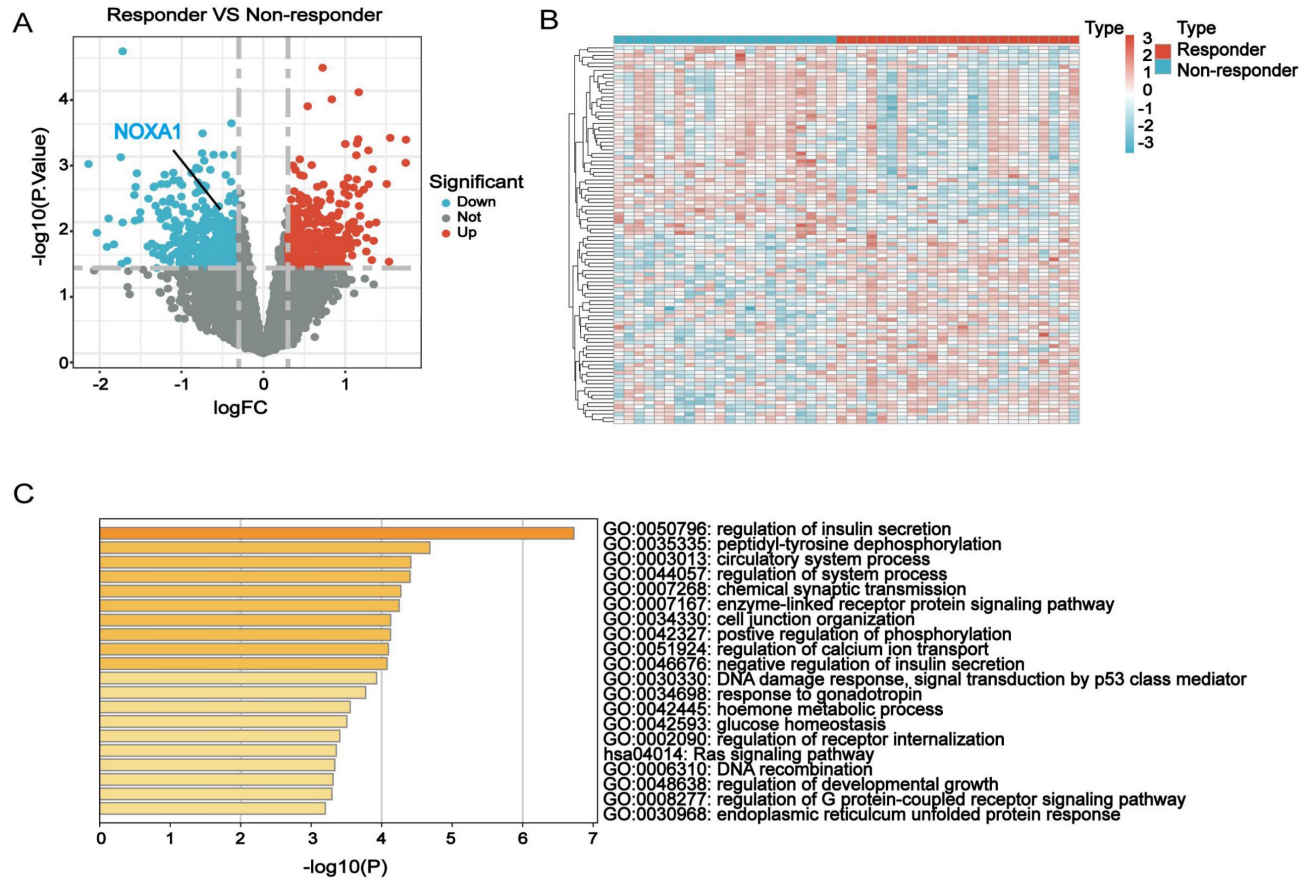
** Identification and functional annotation of RTDEGs in CRC. A-B**: Volcano and heatmap plots showing differentially expressed genes related to radiotherapy resistance in colorectal cancer. **C**: Bar chart indicating GO and KEGG analyses of the differential network.

This study analyzed RTDEGs in CRC cells with diverse radiation responses using the GSE35452 dataset from the GEO database. RTDEGs were determined with a significance level of P<0.05 and an absolute log2 fold change (FC) of at least 0.3. A total of 846 RTDEGs were identified, comprising 423 up-regulated and 423 down-regulated genes. The differences in gene expression were visually represented through volcano and heatmap plots (Figure 1A-B). Metascape pathway enrichment analysis was performed to understand RTDEGs functions. The findings indicated that RTDEGs were predominantly enriched in biological process terms, including DNA damage response, p53-mediated signal transduction, positive regulation of phosphorylation, and DNA recombination (Figure 1C). The results indicate that genes linked to radiation resistance are pivotal in CRC malignant progression, primarily affecting DNA damage response and repair mechanisms.

### Identification of hub genes

**Figure 2:**
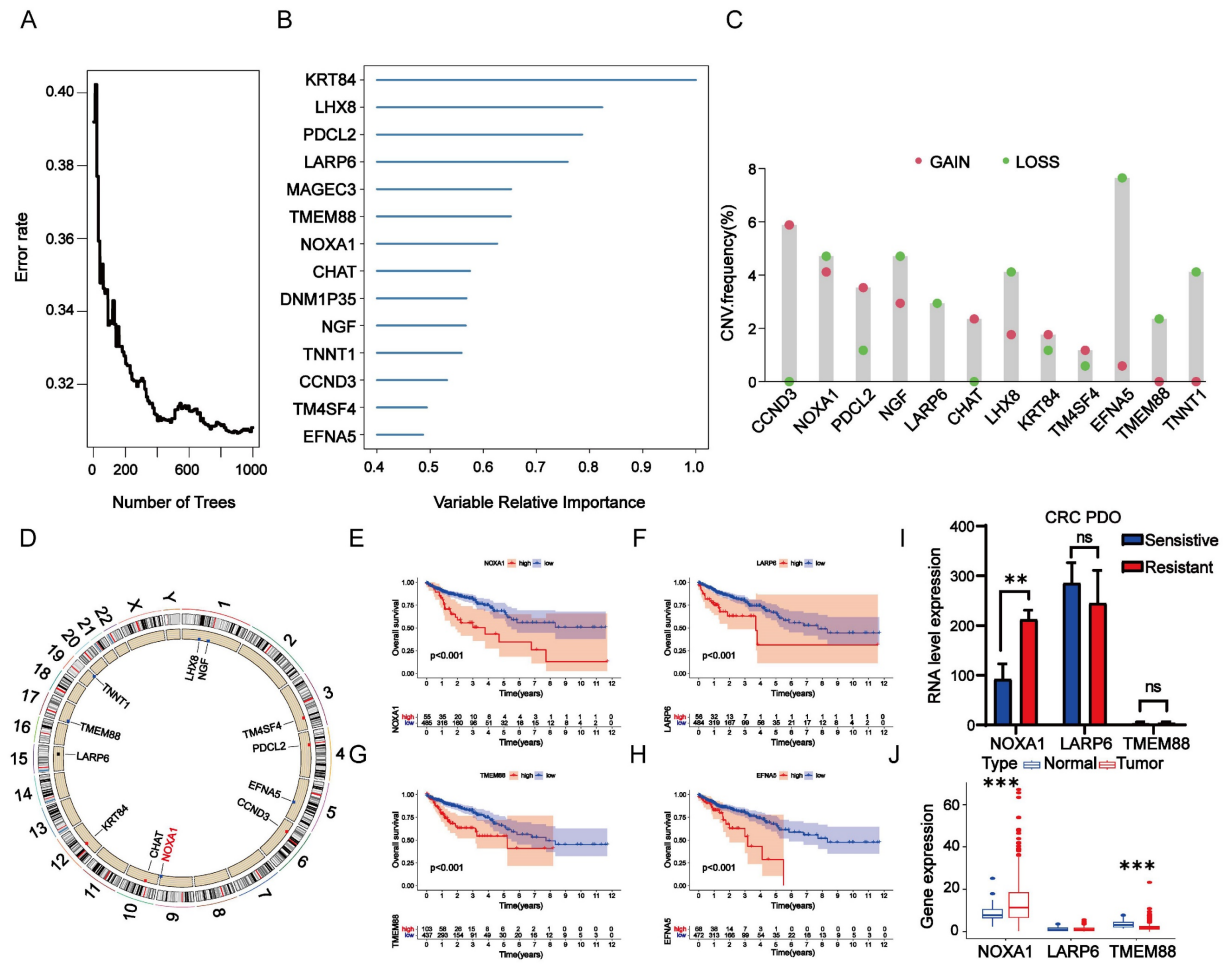
** Analysis of RTDEGs in CRC. A-B**: Random survival forest analysis of RTDEGs. **C-D**: CNV variations in RTDEGs from the TCGA cohort, with green and red dots representing gain and loss of function, respectively. **E-H**: Kaplan-Meier survival analysis showing that low expression of *Noxa1*,* Tmem88*, *Larp6*, and *Enfa5* is associated with better overall survival (P < 0.001). I: Gene expression analysis of DEGs in radiotherapy non-responder and responder CRC organoids. J: Gene expression analysis of DEGs in normal and cancerous colorectal tissues (*P < 0.05).

To identify core genes influencing radiotherapy resistance among RTDEGs, we selected RTDEGs from the TCGA-READ and TCGA-COAD cohorts for random survival forest analysis. Genes with relative importance greater than 0.45 were selected. Ultimately, 14 genes met our screening threshold: *Krt84*, *Lhx8*, *Pdcl2*, *Larp6*, *Magec3*, *Tmem88*, *Noxa1*, *Chat Dnm1p35*, *Ngf*, *Tnnt1*, *Ccnd3*, *Tm4sf4*, and *Enfa5* (Fig. 2A-B). We then conducted a copy number variation (CNV) analysis on these genes. The top 5 genes with the highest CNV frequency were *Enfa5*, *Ccnd3*, *Noxa1*, *Ngf*, and *Lhx8* (except for *Ccnd3*, which has a gain-of-function mutation; the other four have loss-of-function mutations). No relevant mutations were detected in *Magec3* and *Dnm1p35* (Figure 2C-D). Kaplan-Meier survival analysis revealed that low expression of *Noxa1*, *Tmem88*, *Larp6*, and *Enfa5* were significantly linked to improved OS compared to higher expression levels (P<0.001 for each) (Figure 2E-H). While the expression of *Ccnd3* was not statistically significantly associated with the prognosis of colorectal cancer. Therefore, it was excluded from further analysis (Figure S1). Although *Enfa5* expression was linked to the prognosis of colorectal cancer, its variable relative importance was lower than that of other genes. As a result, *Enfa5* was not the main focus of our investigation.

Hence, we then investigated the differential expression of *Noxa1*, *Larp6*, and *Tmem88* in radio-sensitive and radio-resistant organoids. Our findings revealed that *Noxa1* and *Larp6* were notably overexpressed in radio-resistant organoids, while *Tmem88* showed no significant variation. Notably, only *Noxa1*'s differential expression reached statistical significance (Figure 2I). In a comparative analysis, *Noxa1* was primarily expressed in colorectal cancer tissues, *Tmem88* was more prevalent in normal tissues, and *Larp6* showed no significant expression differences between the two (Figure 2J). These findings emphasize the significant role of the *Noxa1* gene in contributing to colorectal cancer's radioresistance.

### Assessment of *Noxa1* as a standalone prognostic indicator

**Figure 3:**
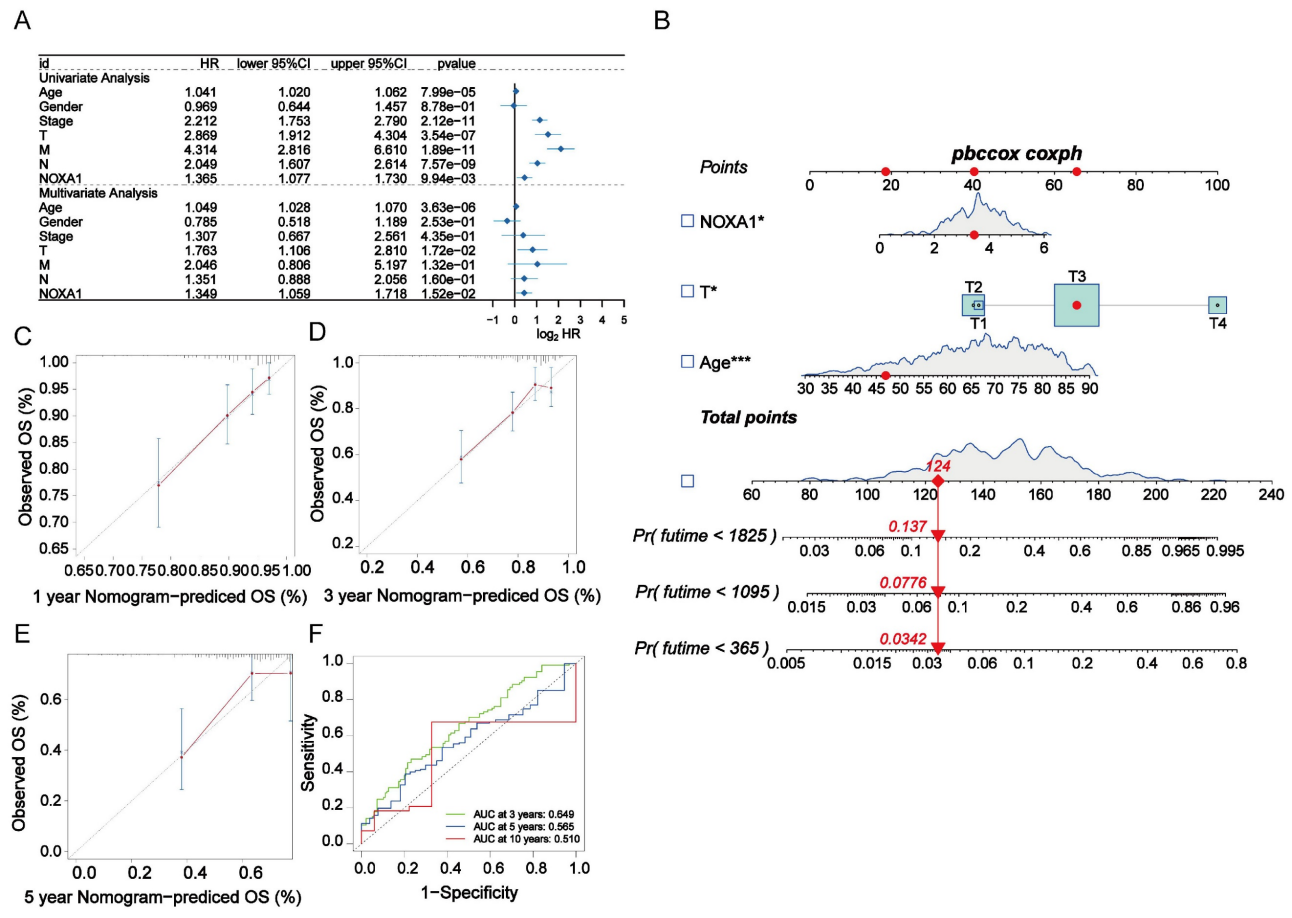
** Construction of the radiotherapy-related prognostic signature in CRC. A**: Forest plot of univariate and multivariate Cox regression analyses for *Noxa1* and clinical characteristics in the TCGA cohort. **B**: Nomogram based on *Noxa1* expression and clinical prognostic factors. **C-E**: Calibration curves predicting 1-, 3-, and 5-year survival rates.** F**: Time-dependent ROC curve for the radiosensitivity-related signature at 3, 5, and 10 years.

Considering the link between elevated *Noxa1* expression and unfavorable outcomes in colorectal cancer patients, along with its loss-of-function mutations, we performed a comprehensive analysis to confirm its role as a standalone prognostic marker. We conducted univariate and multivariate Cox regression analyses to compare *Noxa1* with clinical features such as age, gender, and stages (T, M, N). Our findings revealed that age (P=3.63×10^-6^, HR=1.049), T stage (P=1.72×10^-2^, HR=1.763), and *Noxa1* (P=1.52×10^-2^, HR=1.349) independently predicted colorectal cancer prognosis (Figure 3A). We developed a prognostic nomogram model incorporating these key factors to improve prognostic accuracy (Figure 3B). A calibration plot was used to evaluate the model's precision, revealing strong concordance between predicted and observed overall survival outcomes, thus demonstrating reliable predictive consistency (Figure 3C-E). Additionally, ROC and AUC analyses of the TCGA cohort highlighted *Noxa1*'s potential as a standalone prognostic feature, with AUC values of 0.649 at 3 years, 0.565 at 5 years, and 0.510 at 10 years (Figure 3F), reinforcing *Noxa1*'s significance in predicting long-term outcomes in colorectal cancer patients.

### Overexpression of *Noxa1* enhances radioresistance in CRC

**Figure 4:**
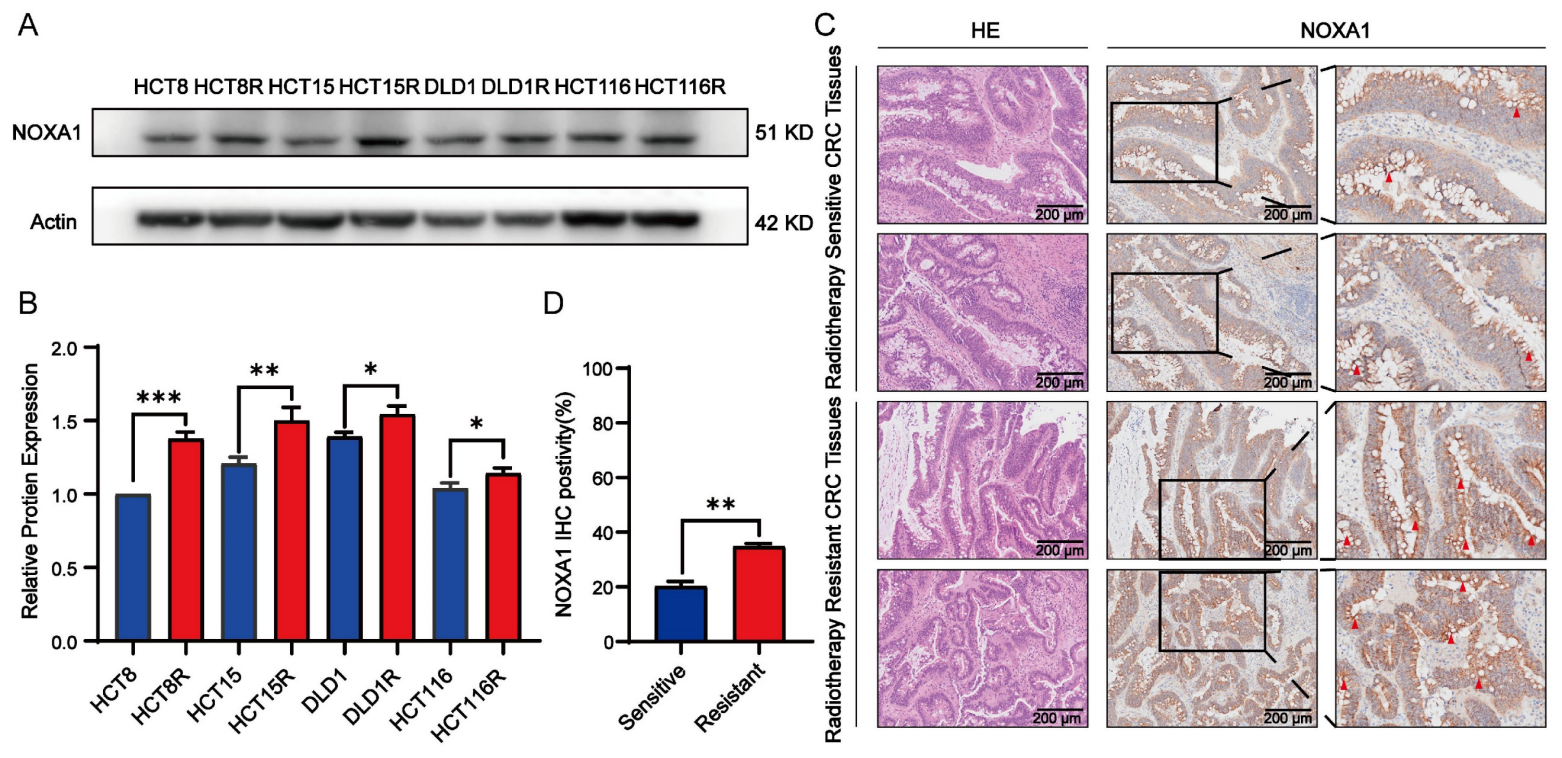
***Noxa1* as a radioresistant oncogene in CRC**.** A-B**: Western blot analysis of CRC cell lines (original blots/gels in Supplementary Fig. 4). **C-D**: H&E and IHC images of radiosensitive vs. radioresistant CRC tissues.

We explored the relationship between *Noxa1* expression and the radioresistance in CRC. Western blot assay indicated that NOXA1 levels increased in various radioresistant CRC cell lines, including HCT8R, HCT15R, DLD1R and HCT116R cells. Notably, HCT15R and HCT8R exhibited higher NOXA1 expression than DLD1R and HCT116R, prompting us to use HCT15R and HCT8R for further experiments (Figures 4A-B).

Additionally, we assessed NOXA1 expression in CRC patient samples, distinguishing between those sensitive and resistant to radiotherapy. Our analysis revealed that NOXA1 expression was markedly elevated in radio-resistant CRC tissues compared to radio-sensitive ones, indicating its potential as a diagnostic marker for CRC radiotherapy sensitivity (Figures 4C-D).

We then exposed HCT8 and HCT15 parental cells to 6Gy radiation. The experimental results showed that radiation did not induce an upregulation of NOXA1 expression in the short term (Figure S2A). On the other hand, we observed that NOXA1 expression was significantly higher in HCT15 cells compared to HCT8 cells, and HCT15 cells exhibited stronger radiation resistance (Figure S2A-B). Based on the expression patterns of *Noxa1* in both radioresistant and radiosensitive cell lines, we speculate that *Noxa1* expression may be closely related to both primary and acquired radiation resistance in colorectal cancer.

**Figure 5:**
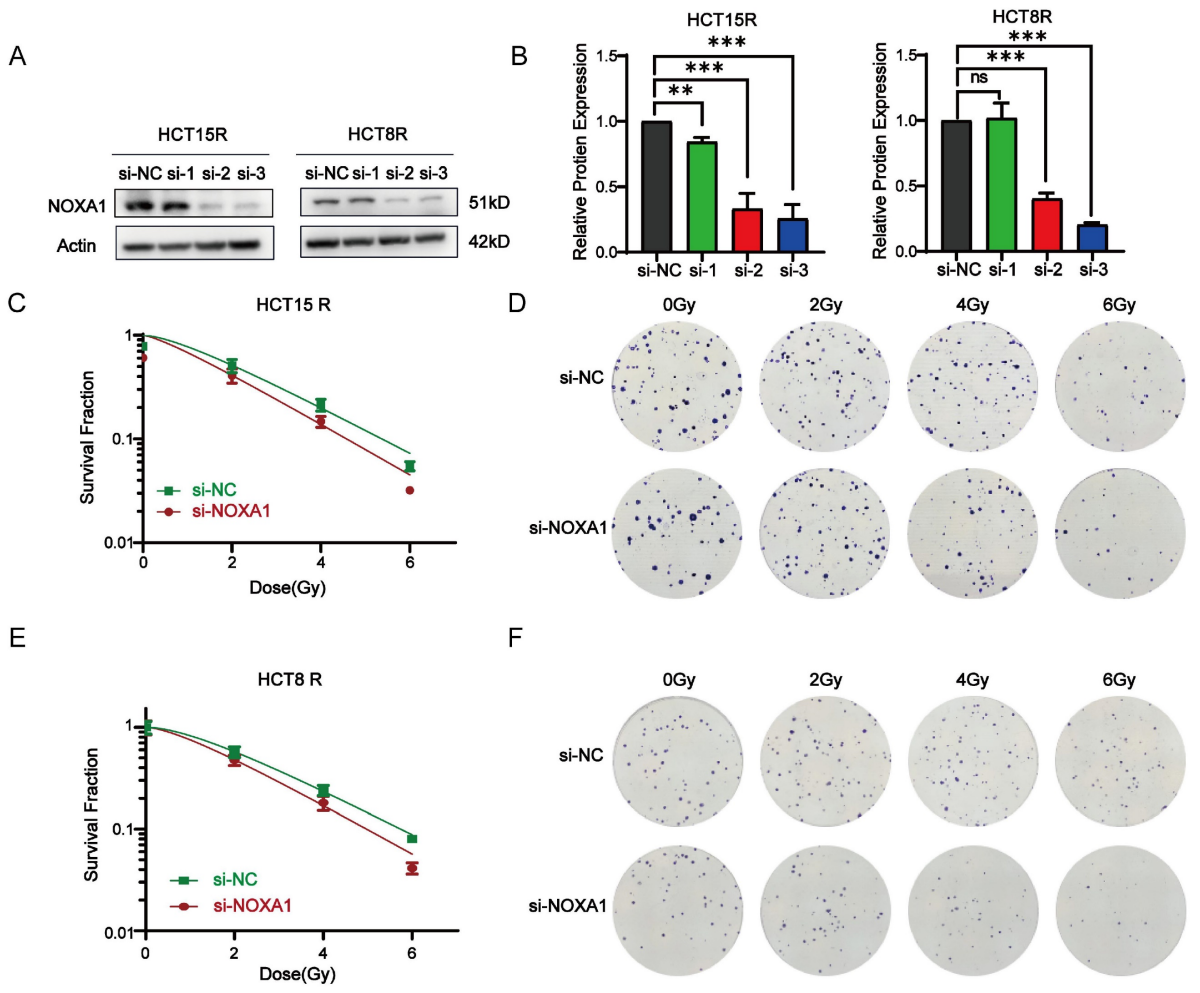
** Impact of *Noxa1* knockdown on radiosensitization in colorectal cancer. A-B**: Western blot analysis of HCT15R and HCT8R cells transfected with si*Noxa1* (original blots/gels in Supplementary Fig. 2). **C-F**: Dose-response survival fractions and colony formation assays for HCT15R and HCT8R cells with/without si*Noxa1* transfection.

To further clarify *Noxa1*'s function, we silence *Noxa1* in HCT15R and HCT8R cells (Figure 5A-B). si*Noxa1*#3 exhibited the highest knockdown efficiency and was selected for further experiments. Knockdown of *Noxa1* led to a marked decrease in the survival fraction of HCT15R and HCT8R cells after radiation exposure (Figures 5C-F).

### *Noxa1* promotes tumor growth in CRC

**Figure 6:**
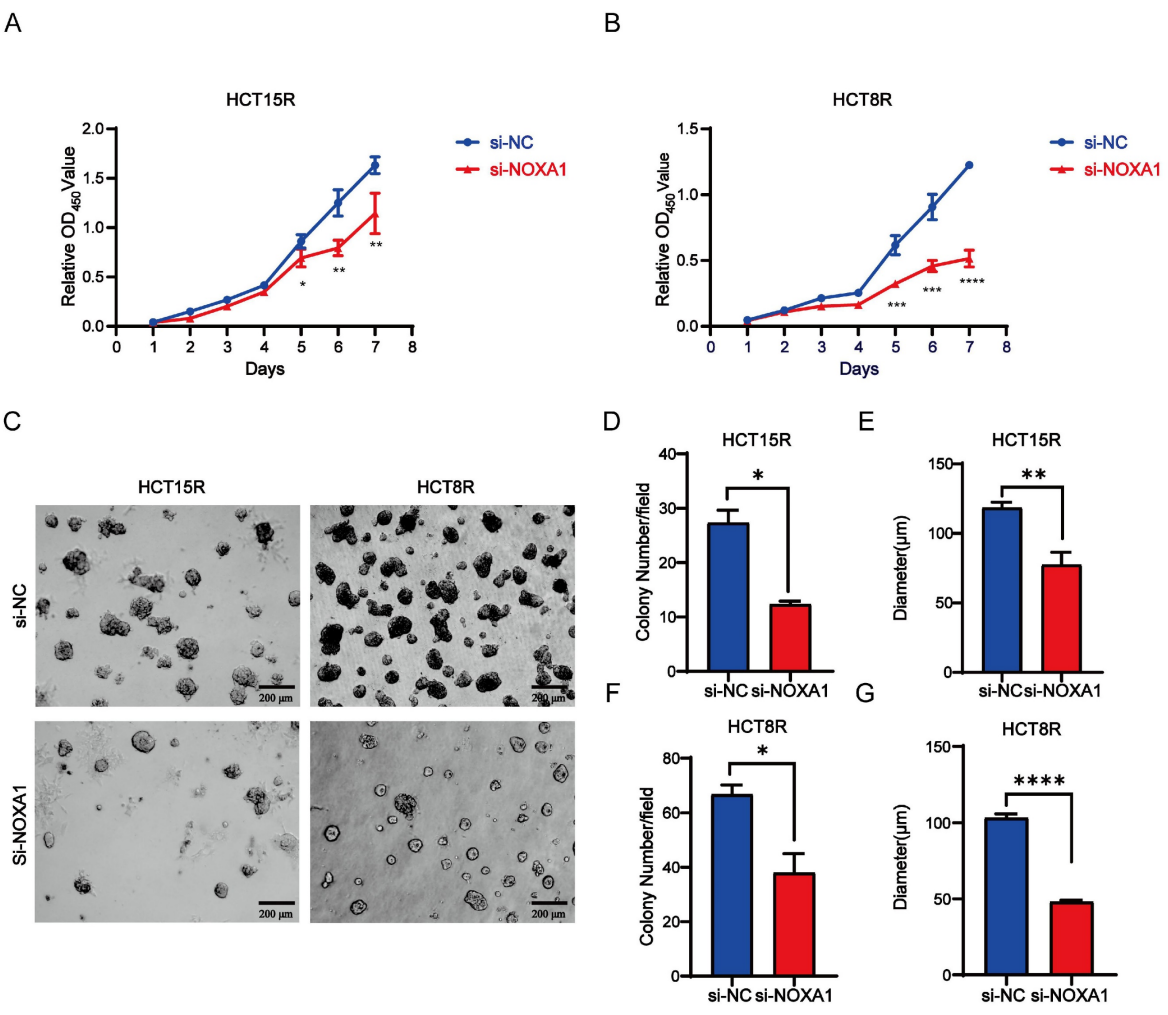
**Noxa1 promotes the occurrence and development of colorectal cancer. A-B**: CCK8 assay of CRC cell viability after *Noxa1* knockdown. **C-G**: The images of three-dimensional (3D) cell culture showed that both the number and diameter of 3D colonies in HCT15R and HCT8R cells with or without si*Noxa1* transfected.

To investigate *Noxa1*'s role in CRC cell viability, we first knocked down *Noxa1* in HCT15R and HCT8R cells and conducted CCK8 assays. *Noxa1* knockdown markedly reduced cell viability (Figures 6A-B). Furthermore, results from three-dimensional (3D) cell culture showed that both the number and diameter of 3D colonies in HCT15R and HCT8R cells transfected with si*Noxa1* were notably decreased, indicating an inhibition of tumor growth capabilities (Figures 6C-G).

### *Noxa1* enhanced radiosensitivity of CRC through ferroptosis pathway

**Figure 7:**
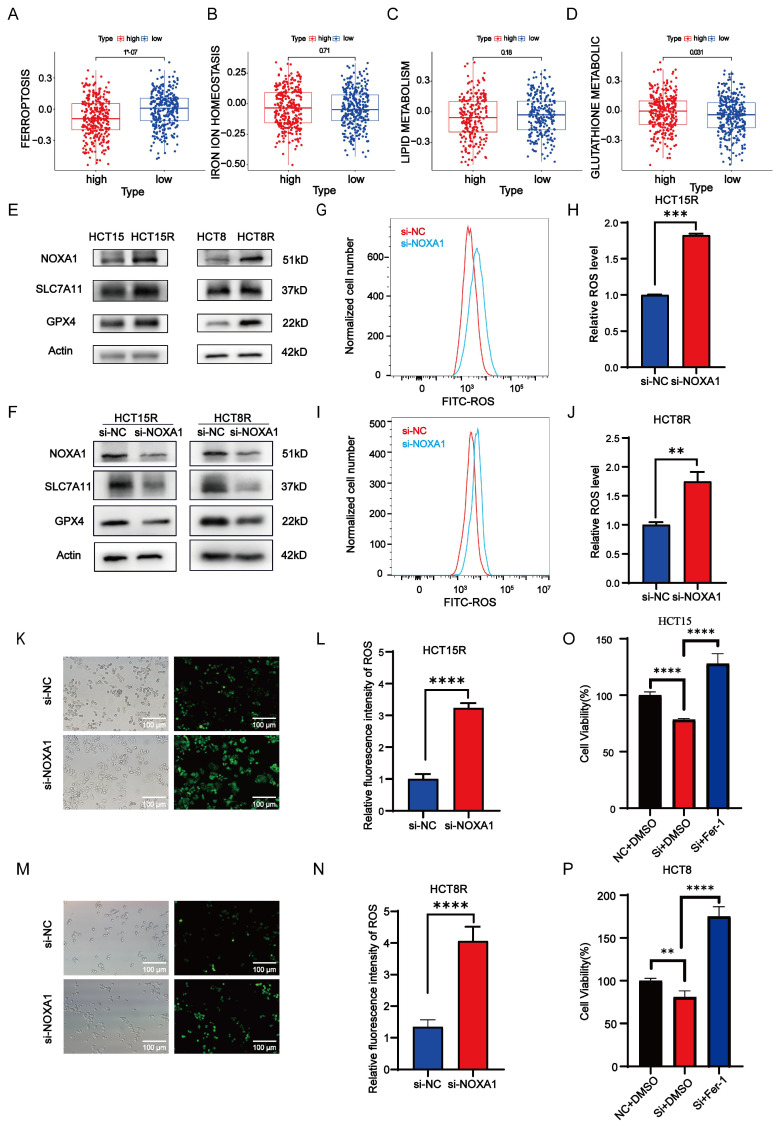
***Noxa1*'s association with ferroptosis in CRC. A-D**: Correlation of Noxa1 expression with ferroptosis, iron homeostasis, lipid, and glutathione metabolism.** E**: Western blot of HCT15, HCT15R, HCT8, and HCT8R cells (original blots/gels in Supplementary Fig. 4). **F**: Western blot of HCT15R and HCT8R cells with/without siNoxa1. **G-N**: Flow cytometry and immunofluorescence assays of ROS in HCT15R and HCT8R cells transfected with si*Noxa1* post-irradiation.** O-P**: CCK8 analysis of *Noxa1* knockdown cells treated with Fer-1.

Ferroptosis, an iron-dependent programmed cell death caused by lipid peroxidation, plays a crucial role in cell death and tumor suppression during radiotherapy. This process encompasses iron metabolism, lipid peroxidation, and glutathione metabolism. Our study identified a negative correlation between *Noxa1* expression and the ferroptosis score, highlighting *Noxa1*'s primary impact on glutathione metabolism pathways (Figure 7A-D).

Western blot assay results showed elevated expression of SLC7A11 and GPX4 in HCT15R and HCT8R cells, alongside increased NOXA1 expression (Figure 7E). Additionally, knockdown of *Noxa1* in HCT15R and HCT8R cells resulted in decreased expression of SLC7A11 and GPX4, suggesting that overexpression of *Noxa1* inhibits ferroptosis in radio-resistant CRC cells (Figure 7F). Furthermore, we measured ROS levels in HCT15R and HCT8R cells and found significantly higher ROS levels in cells transfected with si*Noxa1* (Figures 7G-N), while the ROS levels were significantly higher in the parental cells compared to the radioresistant cells (Figure S3A-D). We then treated *Noxa1-*knockdown cells with Ferrostatin-1 (10µmol). The results showed that cell survival was significantly improved in the *Noxa1*-knockdown cells upon treatment with Ferrostatin-1(Figures 7O-P).

## Discussion

For stage II~III low-to-medium rectal cancer (tumor < 12 cm from the anus) neoadjuvant radiotherapy is the standard treatment^27^. However, due to individual differences, the pathological complete response rate following neoadjuvant chemoradiotherapy for rectal cancer is only 15-27%^28^, and the proportion of patients exhibiting radioresistance ranges from 20% to 40%^29^. This subset of radioresistant colorectal cancer patients experiences poor radiotherapy efficacy due to endogenous cellular resistance or acquired resistance resulting from prolonged radiotherapy^30^. Consequently, we conducted research on radiotherapy-related genes in colorectal cancer. This study identified the radioresistance-associated gene *Noxa1* from the GEO database, revealing that elevated *Noxa1* expression is linked to poor prognosis in colorectal cancer patients. *Noxa1* knockdown suppressed the proliferation of radiotherapy-resistant colorectal cancer cells, indicating its potential role as an oncogene in colorectal cancer development and progression.

In our analyses of colorectal cancer cells, tissues, and organoids, we observed that NOXA1 expression was higher in radioresistant tissues compared to radiosensitive ones. Notably, HCT15R and HCT8R cells exhibited significant radiosensitization following *Noxa1* knockdown after irradiation treatment. The experimental findings robustly indicate that *Noxa1* could serve as a predictive biomarker for radioresistance in CRC.

Radiotherapy damages cell DNA via direct high-energy ionizing radiation and indirectly through ROS generated by radiation hydrolysis. This process leads to cell cycle arrest, ultimately resulting in cell death via apoptosis or necrosis, thereby facilitating tumor cell eradication^31^, ^32^. Consequently, a tumor cell's ability to manage ROS levels significantly influences its radiosensitivity^33^. Enhancing the clearance of intracellular ROS can increase the radiosensitivity of hypoxic tumor cells by disrupting survival signals linked to redox imbalance, thereby protecting cells from ROS damage and facilitating cancer progression^34^. In radiotherapy, elevated NOX levels significantly contribute to ROS production, with tumor cells showing greater NOX expression compared to normal tissues^35^. Therefore, targeting NOXs to effectively downregulate intracellular ROS levels may impact the survival of tumor cells post-ionizing irradiation without affecting normal cells^36^-^40^. NOXA1, as an activating subunit of NOX1, has been demonstrated to influence the production of intracellular ROS. Our measurements indicated that ROS levels were reduced in HCT15R and HCT8R cells compared to HCT15 and HCT8 cells. Research indicates that the upregulation of NOX in cancer cells causes sustained intracellular ROS elevation, facilitating adaptation to elevated ROS and contributing to radiotherapy resistance^41^. Thus, we hypothesize that *Noxa1* overexpression may contribute to radioresistance by chronically elevating ROS production within tumor cells, mediated by antioxidant stress genes induced by the adaptation to high ROS levels.

We employed GSVA to investigate the relationship between *Noxa1* expression and tumor-related signaling pathways, aiming to clarify how *Noxa1* influences radioresistance in colorectal cancer. We discovered that *Noxa1* expression was negatively correlated with ferroptosis, primarily affecting this process through glutathione metabolism. Ferroptosis is negatively regulated by SLC7A11 and GPX4. In HCT15R and HCT8R cells, we observed increased expression of NOXA1, SLC7A11, and GPX4, which inhibited ferroptosis. Following *Noxa1* knockdown, SLC7A11 and GPX4 levels decreased, triggering ferroptosis. Additionally, we found that ROS levels in HCT15R and HCT8R cells significantly increased after *Noxa1* knockdown and subsequent irradiation treatment. High SLC7A11 expression promotes GSH synthesis by mediating cystine transport, upregulating GPX4 activity, enhancing the antioxidant capacity of tumor cells, scavenging ROS, and inhibiting ferroptosiss^42^-^44^. Studies have indicated that radiotherapy reduces the expression of SLC7A11 and GPX4 proteins, thereby triggering ferroptosis^45^. These findings suggest that *Noxa1* might inhibit ferroptosis via upregulating the SLC7A11/GSH/GPX4 axis to eliminate ROS produced by its own overexpression, ultimately leading to radioresistance in colorectal cancer. However, the intrinsic biological regulatory mechanisms linking NOXA1 to the SLC7A11/GSH/GPX4 axis require further investigation.

## Supplementary Material

Supplementary materials and methods, figures.
