# Supplementary material for: NADPH oxidase activator 1 (NOXA1) suppresses ferroptosis and radiosensitization in colorectal cancer
Source: Int J Med Sci. 2025 Feb 18;22(6):1301–12. doi: 10.7150/ijms.107038 (PMC11898851; doi:10.7150/ijms.107038)
Supplement: Supplementary file 1 — Supplementary materials and methods, figures. [file ijmsv22p1301s1.pdf]

Supplementary Material

Material and methods

Colony formation assay

A colony formation assay was conducted to evaluate the radiosensitivity of CRC cells. Cells were plated in 6-well plates at varying densities (100-1200 cells per well) and and exposed to different doses of ionizing radiation (0, 2, 4, 6 Gy), with each condition replicated three times. Following irradiation, the cells were allowed to grow for 14 days to form colonies, which were then fixed and stained with crystal violet.Colonies with over 50 cells were counted to determine the survival fraction, and survival curves were modeled using the single-hit multi-target formula: SF = 1 - (1 - exp(-k*D))^N.

Western Blot (WB) analysis

Total protein from CRC cells was extracted using RIPA lysis buffer (Beyotime Biotechnology) supplemented with 1% protease inhibitors following the manufacturer's instructions. Protein concentrations were determined using the BCA protein assay, and 40μg of total protein was loaded onto a 10% SDS-PAGE gel for separation. The membranes were incubated overnight at 4°C with primary antibodies against NOXA1 (1:1000, Santa Cruz, sc-398873), SLC7A11 (1:1000, CST, 12691S), GPX4 (1:1000, Abcam, ab40993), and Actin (1:5000, HUABIO, EM21002). Following this, the membranes were incubated with secondary antibodies from Jackson ImmunoResearch, USA, at room temperature for 2 hours. Protein bands were visualized using an enhanced chemiluminescence (ECL) kit from Millipore, St. Louis, MO, USA, and the chemiluminescent signals were captured using an imaging system from Azure Biosystems, California. Actin served as the internal standard for total target proteins.

Supplementary Figure

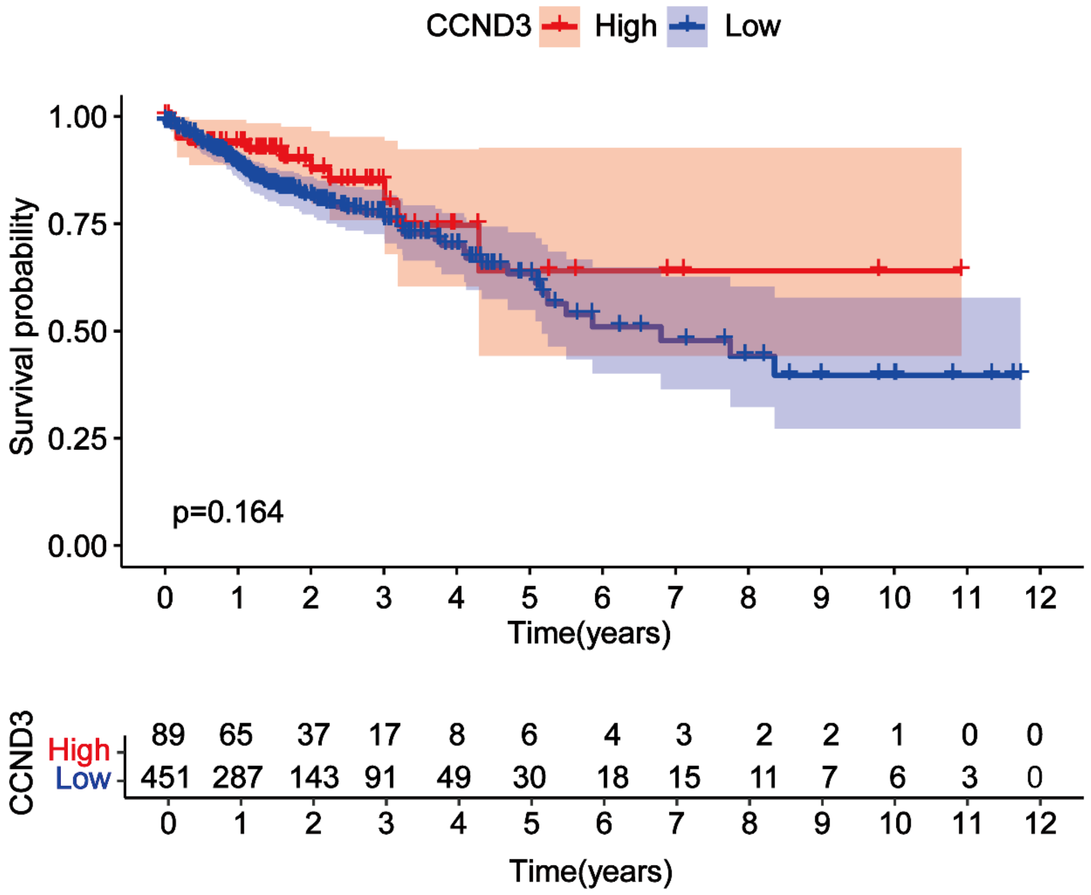

Figure S1. The Kaplan-Meier survival Curve of *Ccnd3*.

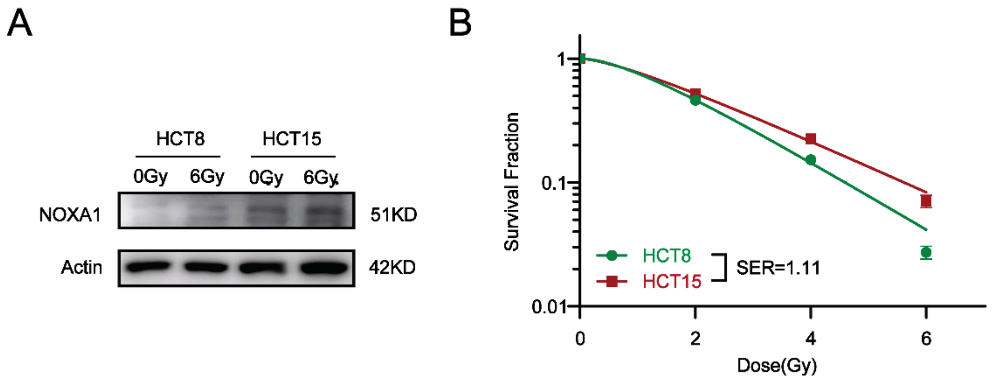

Figure S2. A: Western blot analysis of CRC cell lines exposed to 6Gy irradiation. B: Dose-response survival fractions and colony formation assays for HCT15 and HCT8 cells.

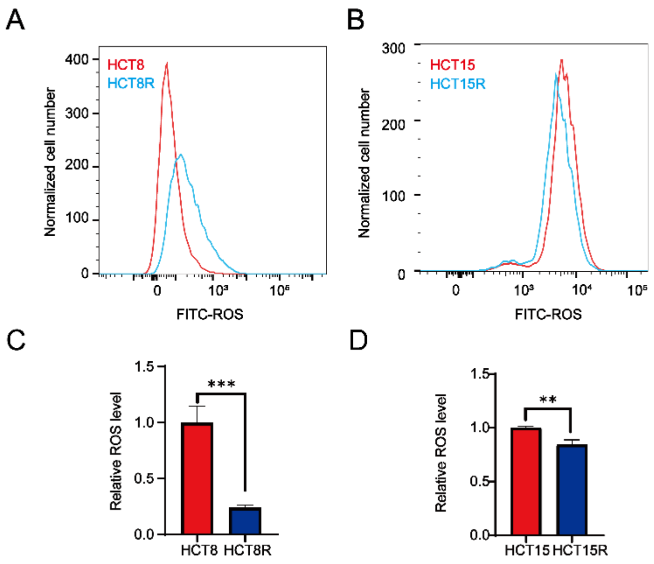

Figure S3. **Flow cytometry assays of ROS in parental and radioresistant cells.**

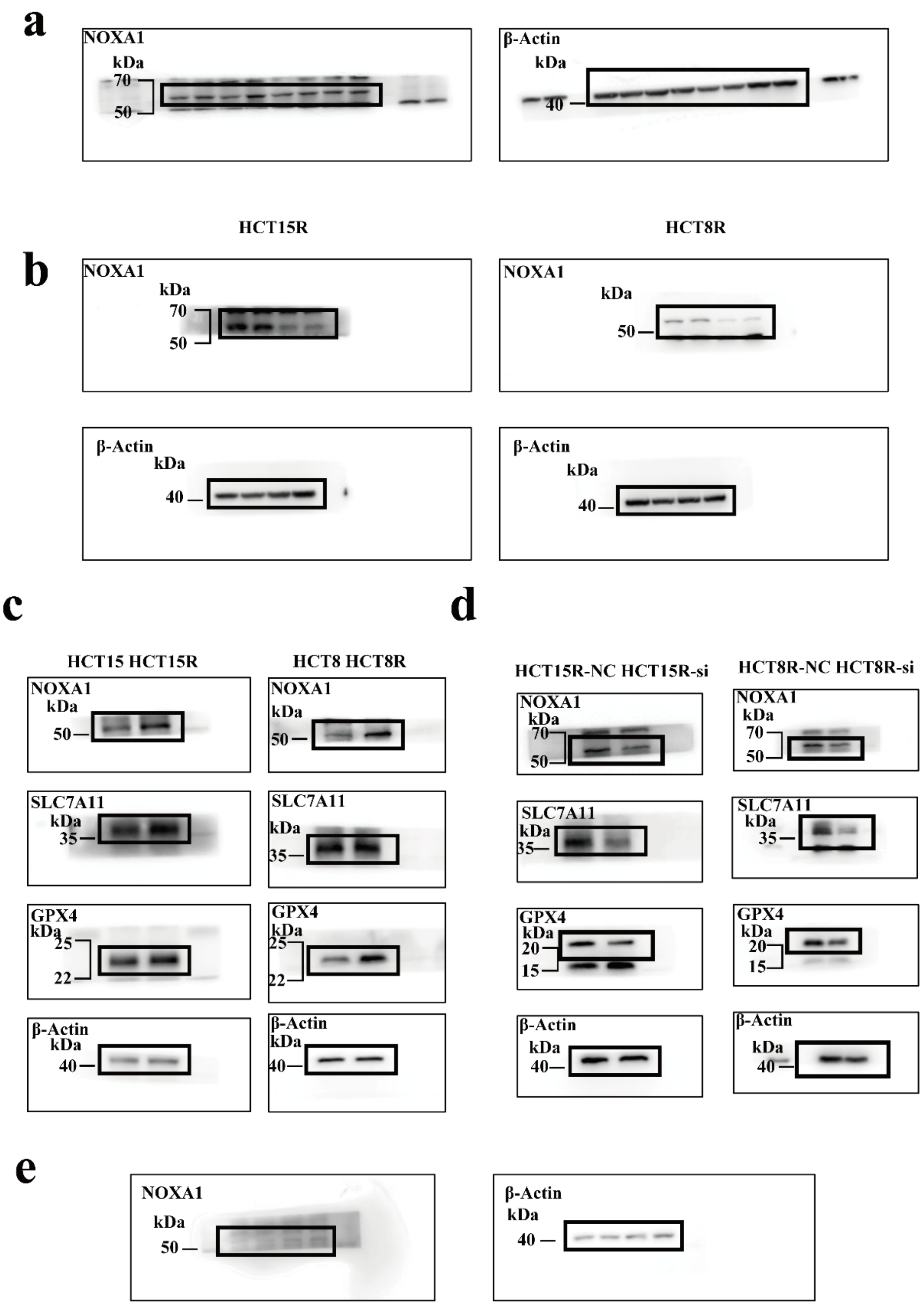

Figure S4. Uncropped blots corresponding to the main figures.

a Uncropped blots for Figure 4A in the main text. **b** Uncropped blots for Figure 5A in the main text. **c** Uncropped blots for Figure 6E in the main text. **d** Uncropped blots for Figure 6F in the main text. **e** Uncropped blots for Supplementary Figure 2A in the supplementary material.
